# Diagnostic and prognostic value of *FOXD1* expression in head and neck squamous cell carcinoma

**DOI:** 10.7150/jca.47978

**Published:** 2021-01-01

**Authors:** Shijie Qiu, Dan Li, Zhisen Shen, Qun Li, Yi Shen, Hongxia Deng, Yidong Wu, Chongchang Zhou

**Affiliations:** 1 Department of Otorhinolaryngology Head and Neck Surgery, Ningbo Medical Center Lihuili Hospital, Ningbo, Zhejiang, China.; 2 Department of Otorhinolaryngology Head and Neck Surgery, Lihuili Hospital affiliated to Ningbo University, Ningbo, Zhejiang, China.; 3 Department of Cardiology, The Second Hospital of Yinzhou, Ningbo, Zhejiang, China.

**Keywords:** *FOXD1*, bioinformatics, head and neck squamous cell carcinoma, prognosis, survival

## Abstract

*FOXD1* has been reported to function as an oncogene in several types of cancer. This study evaluated the expression of *FOXD1* and its role in head and neck squamous cell carcinoma (HNSCC). We mined the Cancer Genome Atlas (TCGA) and Gene Expression Omnibus (GEO) databases for expression profiles, clinical significance, and potential mechanisms of *FOXD1*in HNSCC. Our validation cohort consisted of *FOXD1* mRNA expression in 162 paired HNSCC and adjacent normal tissues, as determined using quantitative real-time polymerase chain reaction. *FOXD1* expression was upregulated in HNSCC in the public databases and in the validation cohort. The expression level of *FOXD1* was associated with DNA amplification and methylation level. The areas under the curves (AUC) of TCGA cohort and the validation cohort were 0.855 and 0.843, respectively. Furthermore, higher *FOXD1* expression was significantly associated with worse overall survival (hazard ratio [HR]: 1.849, 95% confidence interval [CI]: 1.280-2.670, *P* = 0.001) and a lower rate of recurrence-free survival (HR: 1.650, 95% CI: 1.058-2.575, *P* = 0.027) in patients with HNSCC. Moreover, gene set enrichment analysis showed that cases of HNSCC with *FOXD1* overexpression were enriched in bladder cancer, cell cycle, DNA replication, glycosaminoglycan biosynthesis chondroitin sulfate, homologous recombination, glycan biosynthesis, nucleotide excision repair, p53 signaling pathway, pyrimidine metabolism, and spliceosome pathways. In summary, *FOXD1* was significantly upregulated in HNSCC and was a good diagnostic biomarker and an independent predictor of poor survival and low rate of recurrence-free survival in patients with HNSCC.

## Introduction

Head and neck cancer is the sixth most common cancer globally, and is comprised of malignant tumors in the oral cavity, oropharynx, hypopharynx, and larynx. Head and neck squamous cell carcinoma (HNSCC) is the predominant histological type (>90%) of head and neck cancer 1, 2. The International Agency for Research on Cancer reported that the global incidence of HNSCC is more than one million new cases annually, and HNSCC results in 543,000 deaths per year 3. Many studies have shown that tobacco exposure and alcohol consumption are important risk factors for the development of HNSCC 4, 5. Recently, human papillomavirus (HPV) has also been shown to be a strong and independent risk factor for the development of HNSCC 6. Management of HNSCC requires a multi-faceted approach that includes surgery, radiation, and/or chemotherapy. Although substantial progress has been made in treatment of HNSCC, outcomes remain poor, especially in patients with advanced disease. The 5-year survival rate is approximately 50%, which represents only a slight improvement over the last two decades 7. This poor survival rate may be due to late diagnosis, low therapeutic response rates, and high rates of recurrence and metastasis 8. Furthermore, reliable and specific biomarkers for diagnosis and prognosis of HNSCC are lacking. Therefore, it is critical to determine the molecular correlates of HNSCC and to identify reliable biomarkers for diagnosis, prognosis, and monitoring of recurrence, which would allow for improved personalized treatment strategies for patients with HNSCC.

Forkhead box D1 (*FOXD1*), located on chromosome 5q12, belongs to the forkhead box transcription factor family, and is involved in numerous physiological processes and biological functions, such as embryonic development and organogenesis, cell cycle regulation, control of metabolism, stem cell niche maintenance, and signal transduction 9, 10. *FOXD1* was identified and described for the first time in the forebrain neuroepithelium and is considered an important factor during retinal development 11. Furthermore, *FOXD1* was found to be a mediator of successful progression of cell reprogramming through self-renewal and differentiation 12, 13. Recent studies showed that *FOXD1* was associated with carcinogenesis, tumor progression, and metastasis in numerous cancers 14-16. *FOXD1* was reported to be overexpressed in colorectal cancer tissues, and expression levels correlated with tumor size, differentiation, tumor node and metastasis (TNM) stage, lymph node metastasis, and poor prognosis 17. *FOXD1* has also been shown to be highly expressed in non-small cell lung cancer, and promoted cell proliferation, migration, invasion, and metastasis through activation of Vimentin 18. Zhao et al. found that *FOXD1* was up-regulated in breast cancer tissues and promoted cell proliferation and chemotherapeutic drug resistance by targeting p27 expression 19. These findings suggested that *FOXD1* may function as an oncogene in several cancers. However, the role of *FOXD1* in HNSCC has not been characterized.

In the present study, we evaluated *FOXD1* mRNA expression in HNSCC using data from The Cancer Genome Atlas (TCGA) database and the Gene Expression Omnibus (GEO) database. We also investigated the association of *FOXD1* expression with clinicopathological parameters and evaluated its potential as a diagnostic biomarker of HNSCC. Furthermore, we assessed the prognostic value of *FOXD1* for overall survival (OS) and recurrence-free survival (RFS). We performed gene set enrichment analysis (GSEA) to identify *FOXD1* related signaling pathways involved in tumorigenesis and progression of HNSCC. In addition, tissue samples from patients for whom clinicopathological and survival data were available were analyzed to validate the results of the bioinformatic analysis.

## Materials and methods

### Mining analysis using the Cancer Genome Atlas (TCGA) dataset

Level-3 *FOXD1* RNA-seq data consisting of 520 HNSCC tissues and 44 normal controls were downloaded from the University of Santa Cruz Xena browser (https://xenabrowser.net, up to June 11, 2019). Related clinicopathological data including sample type, age at initial pathologic diagnosis, gender, histological types, smoking history, alcohol history, anatomic neoplasm subdivision, HPV status by p16 testing, perineural invasion present, histologic grade pathologic T, pathologic N, pathologic stage, OS status, OS time, RFS status, RFS time, DNA methylation, and gistic2 threshold-processed copy-number alteration (-1: copy deletion; 0: no change; +1: amplification) were obtained for secondary analysis. The β values of cg23454038 probes mapping 200 bp downstream of the transcription start sites of *FOXD1* were defined as the *FOXD1* promoter methylation level.

### Mining analysis using the Gene Expression Omnibus (GEO) dataset

The keywords “head and neck squamous cell carcinoma” were used to search the GEO database (https://www.ncbi.nlm.nih.gov/gds/), and *FOXD1* expression profiles from GSE6631 19 were downloaded. The platform for GSE6631 was GPL8300, [HG_U95Av2] Affymetrix Human Genome U95 Version 2 Array, which contains twenty-two paired head and neck squamous cell carcinoma samples and adjacent normal tissue.

### Clinical sample collection

One hundred sixty-two surgical specimens from patients with HNSCC, including tumor and adjacent normal tissues, were collected (125 male and 37 female, age 38‑79 years; average age 61.7‑years of age). The patients were diagnosed based on clinical features and histopathological examination at the Ningbo Medical Centre Lihuili Hospital (Ningbo, China) and the Affiliated Tumor Hospital of Xiangya Medical School (Changsha, China) from February 2014 to November 2018. Upon removal, specimens were immediately placed in RNA-fixer Reagent (Bioteke, Beijing, China) and stored at -80 °C until be used in experiments. None of the patients underwent radiation or chemotherapy prior to surgery. Histological type was identified independently by two experienced pathologists. Clinicopathological features were collected from medical records, and tumor stages were classified according to the 8th Edition HNSCC TNM staging system of the American Joint Committee on Cancer (AJCC) 20. During the follow-up period, 11 patients were censored, and 72 patients died. The median patient follow-up time was 27.2 months. Overall survival time was defined as the period between pathological diagnosis and death. This study was approved by the Ethics Committee of the Ningbo Medical Centre Lihuili Hospital and the Affiliated Tumor Hospital of Xiangya Medical School. All patients provided written informed consent.

### Total RNA preparation and real-time quantitative reverse transcription polymerase chain reaction (qRT-PCR)

Total RNA was extracted from specimens using TRIzol reagent (Invitrogen, Carlsbad, CA, USA), then reverse transcribed into cDNA using GoScript Reverse Transcription (RT) System (Promega, Madison, WI, USA) following the manufacturer's instructions. Quantitative RT-PCR was performed using GoTaq qPCR Master Mix (Promega, Madison, WI, USA) on a LightCycler 480 real-time PCR System (Roche, Basel, Switzerland). Glyceraldehyde-3-phosphate dehydrogenase (*GAPDH*) was used as an internal reference. The primers were synthesized by Huada Biotech Ltd. (Shen Zhen, China). The specific primer sequences in the experiment were as follows: *FOXD1:* 5′-TGAGCACTGAGATGTCCGATG-3′ (forward primer) and 5′-CACCACGTCGATGTCTGTTTC-3′ (reverse primer), *GAPDH*: 5′-CCATGGAGAAGGCTGGGG-3′ (forward primer) and 5′- CAAAGTTGTCATGGATGACC -3′ (reverse primer). The thermal cycling program was as follows: 95 °C for 10 min, then 45 cycles at 95 °C for 20 s, 57 °C for 35 s, and 72 °C for 30 s. Cycle threshold (Ct) values were recorded, and all results were expressed as means of three independent experiments. *FOXD1* expression levels were analyzed using the 2^-ΔCt^ method 21.

### Survival analysis

Patients with HNSCC for whom *FOXD1* expression and survival data were collected were divided into 2 groups (low and high *FOXD1* expression) based on the maximum Youden index of the receiver operating characteristic (ROC) curves for death and recurrence. Overall survival and RFS were compared between the high and low *FOXD1* expression groups using Kaplan-Meier analysis with the log-rank test. Univariate and multivariate Cox proportional hazards models were performed to evaluate the relative risk factors associated with OS or RFS, and hazard ratios (HR) with 95% confidence intervals (CI) were obtained for each variable. Only significant factors in the univariate analysis were included in the multivariate analysis.

### Gene set enrichment analysis (GSEA) resulted in identification of *FOXD1*-related signaling pathways in HNSCC

Gene set enrichment analysis was performed to identify potential mechanisms of *FOXD1* in development of HNSCC. Samples from TCGA were divided into high and low *FOXD1* expression groups based on the median *FOXD1* expression. MsigDB Collection (h.all.v6.2.symbols.gmt) was used as a reference for GSEA. Significantly enriched pathways related to tumor biological process were selected according to normalized enrichment score (NES) with 1000 permutations. A pathway was regarded as significantly enriched when the *P*-value was less than 0.05.

### Statistical analysis

Statistical Program for Social Sciences (SPSS) 20.0 software (SPSS Inc., Chicago, IL, USA) and R 3.1.2 software (https://www.r-project.org/) were used to perform statistical analysis and to generate figures. Between-group comparisons of *FOXD1* expression and the correlation between *FOXD1* expression and clinicopathological features were performed using independent or paired Student's t-tests, as appropriate. Receiver operating characteristic curves were generated, and areas under the curves (AUC) were calculated to determine the diagnostic power of *FOXD1* in HNSCC. The cut-off point was defined as the maximum Youden index. Pearson correlation coefficients were generated to evaluate the association between *FOXD1* mRNA expression and *FOXD1* DNA methylation levels. *P* ≤ 0.05 was considered statistically significant.

## Results

### *FOXD1* was upregulated in patients with HNSCC

**Figure 1:**
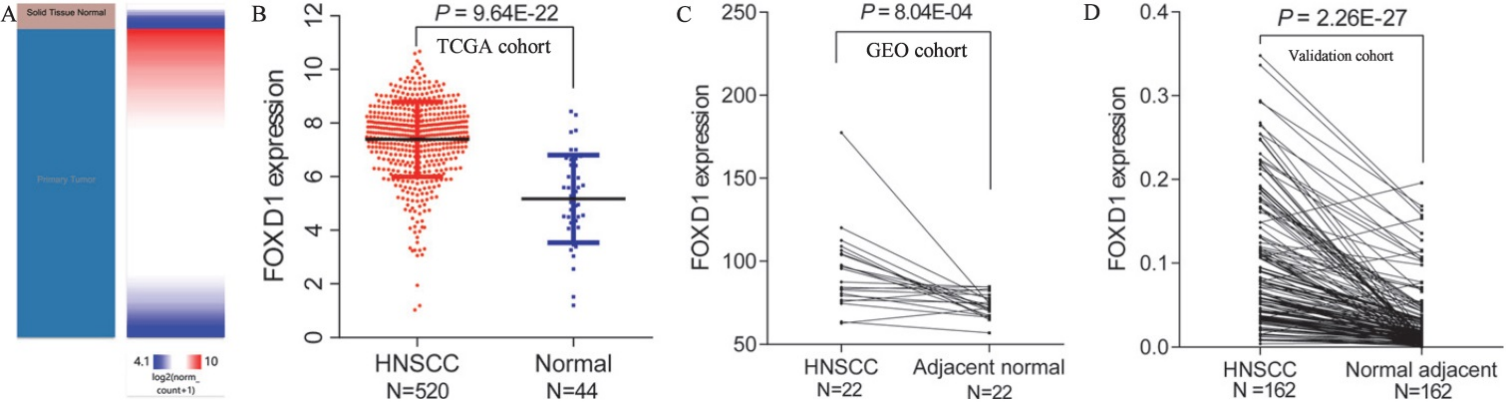
** Differential expression of *FOXD1* in HNSCC and normal tissue in TCGA and GEO databases.** The heatmap (A) and plot (B) showed that HNSCC tissues (N=520) had significantly elevated *FOXD1* expression levels compared with those in normal tissues (N=44) in TCGA database. *FOXD1* expression was higher in HNSCC tissues than in adjacent normal tissues in the GEO database (C).* FOXD1* was significantly overexpressed in HNSCC tissues compared with adjacent normal tissues in the validation cohort (D).

Using Xena browser, we reviewed *FOXD1* mRNA expression in 520 HNSCC and 44 normal tissues in TCGA database. The results indicated that *FOXD1* mRNA expression was significantly higher in HNSCC tissues than that in normal tissues (*P* = 9.64E-22, Figure 1A and B). Similarly, *FOXD*1 was significantly upregulated in one dataset (containing 22 pairs of HNSCC and adjacent normal tissues) obtained from the GEO database (*P* = 8.04E-4, Figure 1C). To further investigate the expression of *FOXD*1 in HNSCC, we collected HNSCC tissue and adjacent normal tissue from 162 patients with HNSCC for use as a validation cohort. Quantitative RT-PCR analysis showed that *FOXD*1 expression was significantly higher in HNSCC tissues than that in normal tissues (*P* = 2.26E-27, Figure 1D).

### Relationship between *FOXD1* expression levels and clinicopathological factors

We examined the correlation between *FOXD1* expression and clinicopathological features of patients with HNSCC. In the TCGA cohort, we found that patients with HNSCC who did not smoke, and had tumors located in the larynx, had significantly elevated *FOXD1* expression. However, *FOXD1* expression was not associated with age, gender, alcohol history, histologic grade, HPV status, perineural invasion, tumor category, nodal category, or pathologic stage (Table ​1). Furthermore, *FOXD1* expression was not significantly associated with any clinicopathological factors in the validation cohort (Table 2).

### The diagnostic value of *FOXD1* for HNSCC

**Figure 2:**
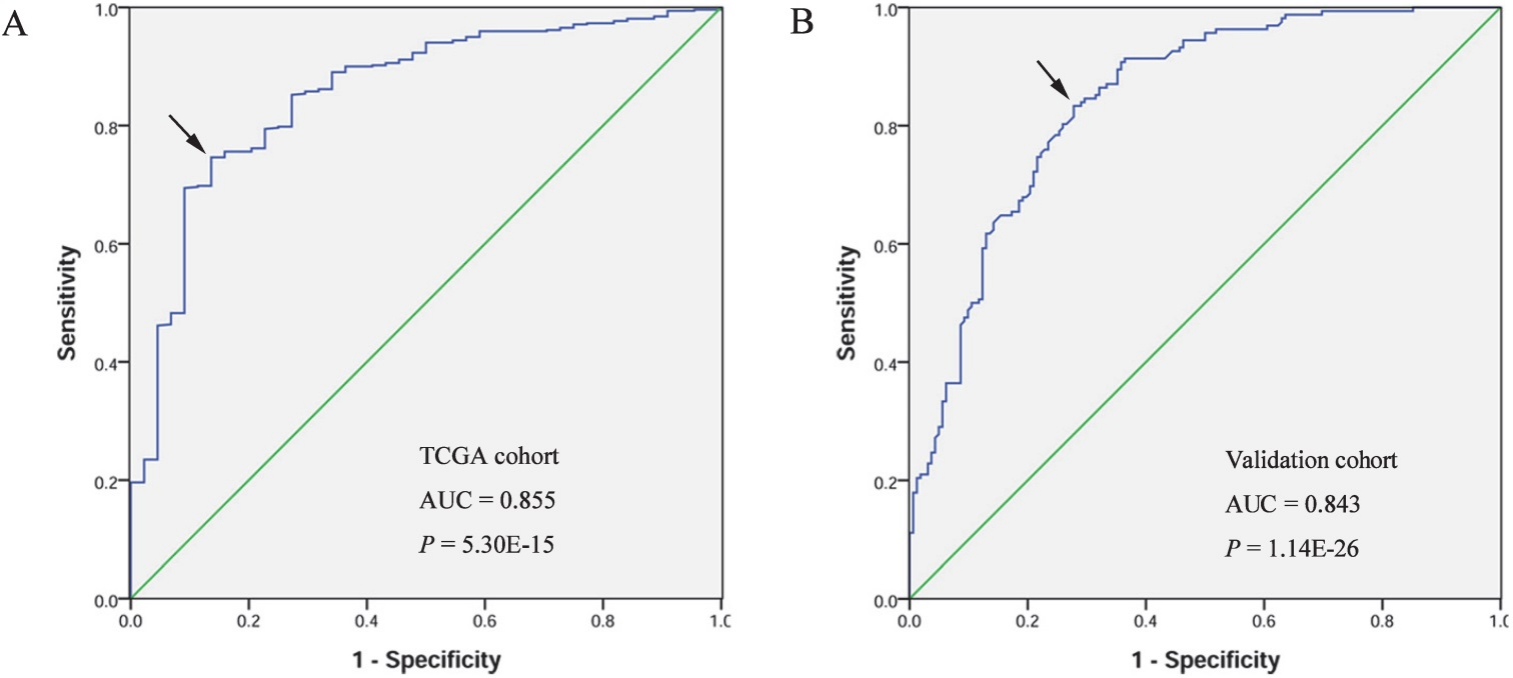
** Diagnostic value of *FOXD1* expression in patients with HNSCC.** Receiver operating characteristic curve of *FOXD1* in HNSCC from TCGA cohort (A). Receiver operating characteristic curve of *FOXD1* in HNSCC in the validation cohort (B). AUC: area under the curve.

A receiver operating characteristic (ROC) curve was generated and the area under the ROC curve (AUC) was calculated to evaluate the diagnostic value of *FOXD1*. The AUC of TCGA cohort was 0.855, with sensitivity and specificity values of 0.746 and 0.864, respectively. In the validation cohort, the AUC was 0.843, with sensitivity and specificity values of 0.833 and 0.722, respectively (Figure 2).

### High *FOXD1* expression was an independent risk factor for poor OS in patients with HNSCC

**Figure 3:**
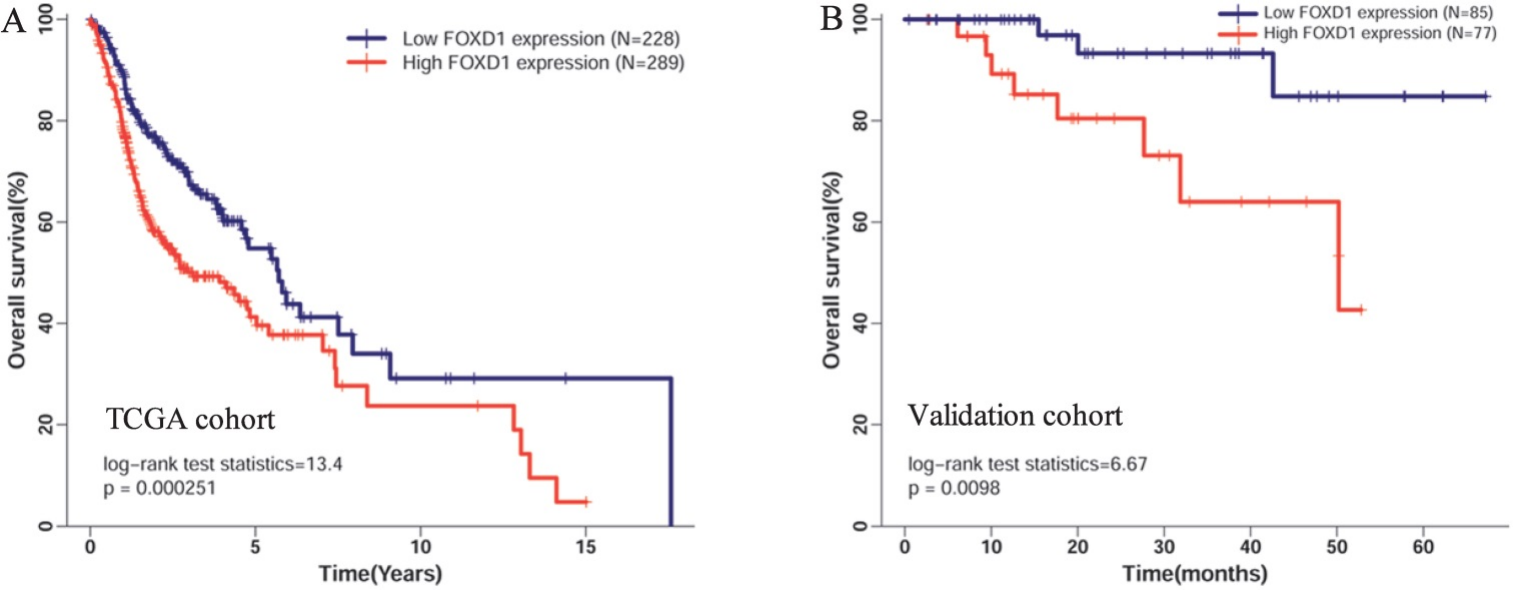
**Kaplan-Meier curves of overall survival in patients with HNSCC with high and low* FOXD1* expression in (A) the TCGA cohort and (B) the validation cohort.** Overall survival time in the high *FOXD1* expression group was shorter than that in the low expression group in TCGA database (Log-rank *P* = 2.51E-4). The Kaplan-Meier curve of the validation cohort showed that high *FOXD1* expression was associated with significantly worse OS (Log-rank *P* = 0.01).

Five hundred seventeen patients with complete *FOXD1* expression and overall survival data in TCGA cohort were divided into high expression (289 patients) and low expression groups (228 patients) according to the maximum Youden index of the ROC curve for death. The Kaplan-Meier curve showed that high mRNA expression of *FOXD1* was associated with poor overall survival rate (Figure 3A, log-rank *P* = 2.51E-4). To further assess the prognostic value of *FOXD1*, we collected follow-up information from 162 patients with HNSCC. These results showed that patients with high *FOXD1* expression had worse overall survival than patients with lower *FOXD1* expression (Figure 3B, log-rank *P* = 0.0098). Subsequently, a Cox proportional hazard regression model was used to screen prognostic factors for patients with HNSCC in TCGA database. As shown in the Table 3, univariate analysis showed that older age (HR = 1.318, 95% CI: 1.003-1.731, *P* = 0.045), female (HR = 1.349, 95% CI: 1.014-1.796, *P* = 0.040), positive perineural invasion (HR = 2.135, 95% CI: 1.516-3.007, *P* = 1.42E-05), advanced pathologic stage (HR = 1.754, 95% CI: 1.203-2.558, *P* = 0.004), and high *FOXD1* expression (HR = 1.665, 95% CI: 1.264-2.194, *P* = 2.93E-04) were significantly associated with poor OS in patients with HNSCC. Using significant factors identified in the univariate analysis, multivariate analysis confirmed that high *FOXD1* expression (HR = 1.849, 95% CI: 1.280-2.670, *P* = 0.001), positive perineural invasion (HR = 2.051, 95% CI: 1.436-2.931, *P* = 7.90E-05), and advanced pathologic stage (HR = 1.739, 95% CI: 1.051-2.877, *P* = 0.31) were independent risk factors for poor OS in patients with HNSCC.

### High *FOXD1* expression was an independent factor for poor RFS in patients with HNSCC patients

**Figure 4:**
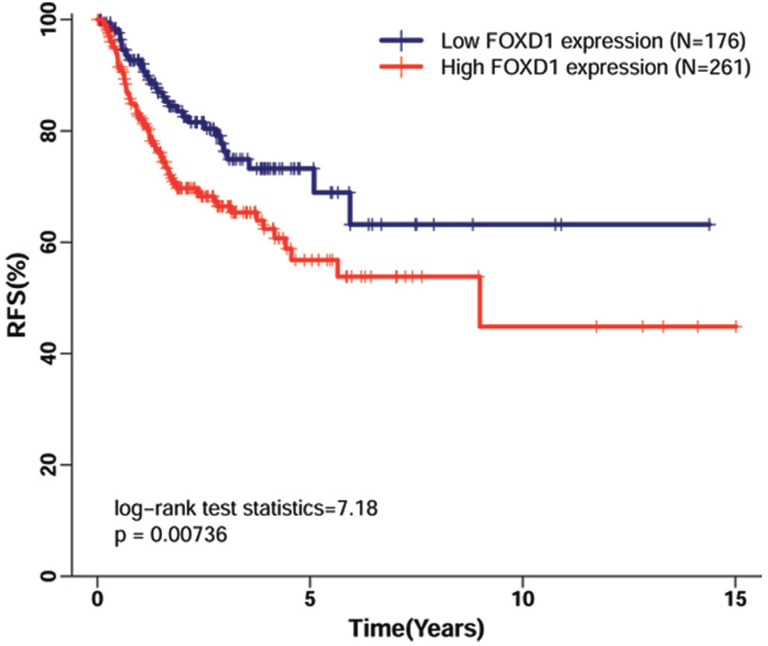
** Kaplan-Meier curves of recurrence-free survival in patients with HNSCC with high and low *FOXD1* expression.** The Kaplan-Meier curves showed that high *FOXD1* expression predicted reduced rate of recurrence-free survival in patients with HNSCC (Log-rank *P* = 0.007).

Using TCGA dataset, which contained follow-up data for HNSCC recurrence in 438 patients, we evaluated the association of *FOXD1* expression with RFS in patients with HNSCC. The Kaplan-Meier curve for RFS showed that high *FOXD1* expression was associated with poor RFS (Figure 4, log-rank *P* = 0.007). This finding was further confirmed by univariate Cox proportional hazard analysis (Table 4), in which patients with high *FOXD1* expression in tumors were at significantly increased risk of recurrence compared to patients with low *FOXD1* expression in tumors (HR = 1.725, 95% CI: 1.152-2.583, *P* = 0.008). Subsequently, multivariate analysis showed that high *FOXD1* expression was an independent risk factor for RFS in patients with HNSCC (HR = 1.650, 95% CI: 1.058-2.575, *P* = 0.008) after adjustment for significant prognostic clinicpathological parameters (smoking history and pathologic stage).

### *FOXD1* expression was related to DNA copy number alteration and promoter methylation in HNSCC

**Figure 5:**
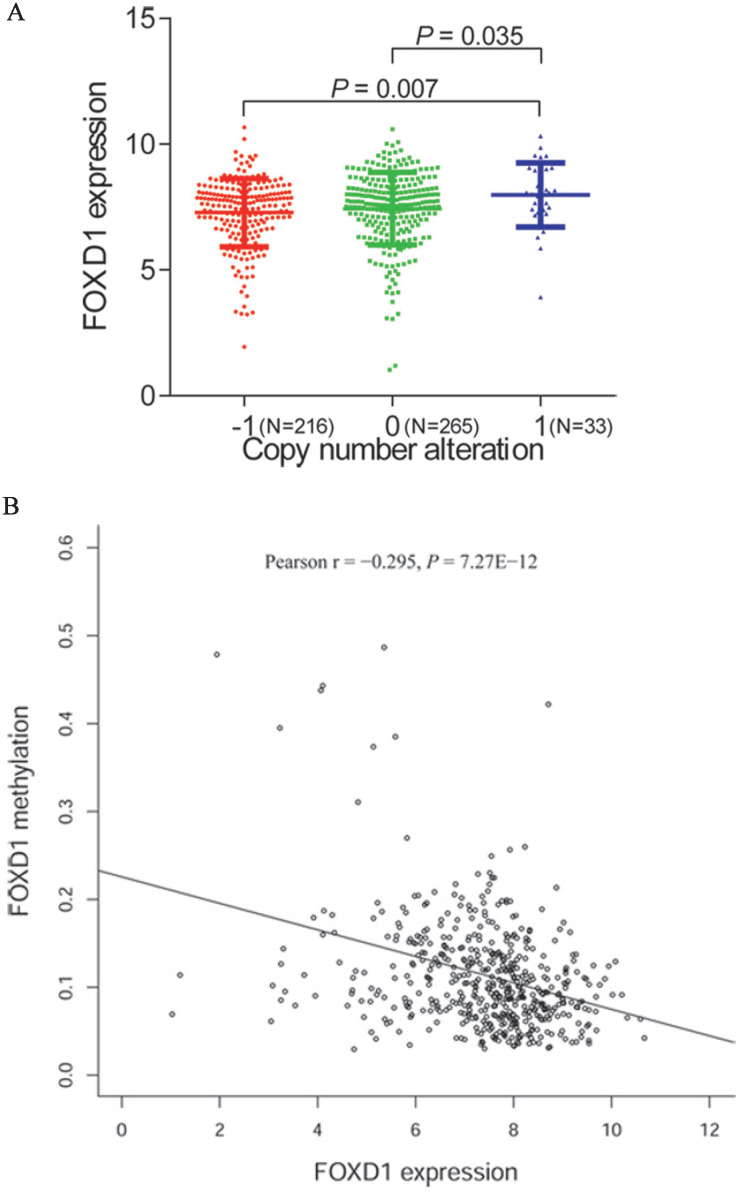
***FOXD1* expression may be modulated by changes in DNA copy number and DNA methylation.** Comparison of *FOXD1* RNA expression based on copy number (A). *FOXD1* RNA expression was significantly negatively correlated to promoter methylation levels (Pearson r = -0.295, *P* = 7.27E-12). -1: copy deletion; 0: no change; +1: amplification.

To further investigate the mechanism of *FOXD1* overexpression in HNSCC, we examined the association of *FOXD1* expression with copy number alterations and promoter methylation using TCGA database. Among 514 patients for whom *FOXD1* DNA copy number was determined, 33 cases (14.5%) showed DNA amplification (+1) and 216 cases (31.8%) showed copy deletion (-1). We showed that DNA amplification was associated with increased *FOXD1* expression (*P* = 0.007, compared to the copy deletion group, Figure 5A). Subsequently, we observed a negative correlation (Person r = -0.295, *P* = 7.27E-12) between *FOXD1* expression and promoter methylation (Figure 5B).

### GSEA identified *FOXD1*-related signaling pathways in HNSCC

**Figure 6:**
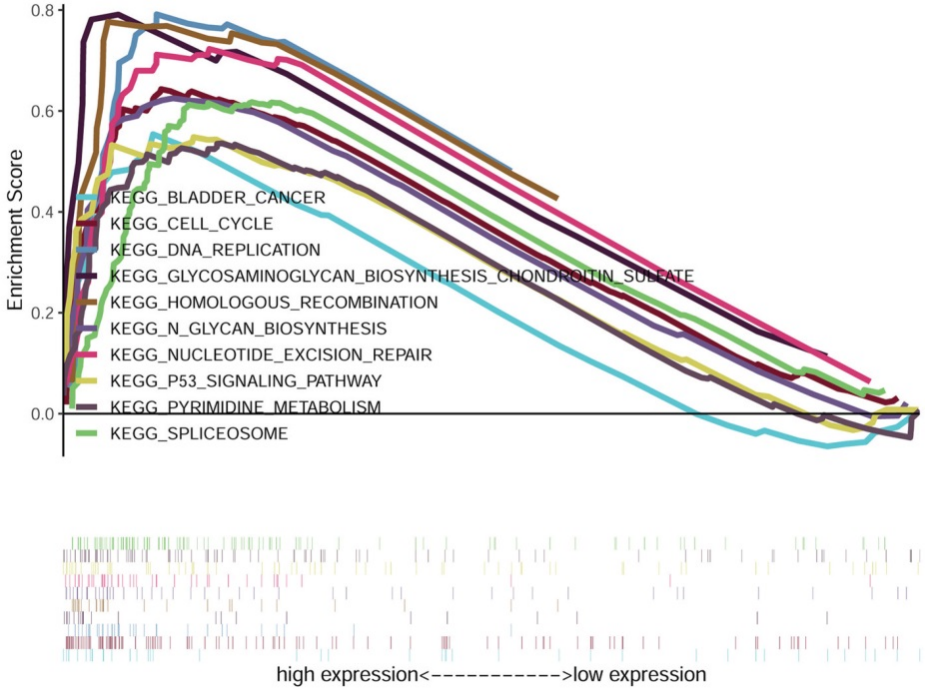
**Gene set enrichment analysis (GSEA) of HNSCC samples in TCGA dataset.** The results of GSEA showed that bladder cancer, cell cycle, DNA replication, glycosaminoglycan biosynthesis chondroitin sulfate, homologous recombination, glycan biosynthesis, nucleotide excision repair, p53 signaling pathway, pyrimidine metabolism, and spliceosome were enriched in the *FOXD1* overexpression group.

We compared the low and high *FOXD1* groups using GSEA analysis to identify* FOXD1*-related signaling pathways activated in HNSCC. The results showed significant differences in gene sets (false discovery rate [FDR] *P*-value *<* 0.05) in the enrichment of MSigDB Collection (h.all.v6.2.symbols.gmt). Detailed results are provided in Table 5. The results showed that bladder cancer, cell cycle, DNA replication, glycosaminoglycan biosynthesis chondroitin sulfate, homologous recombination, glycan biosynthesis, nucleotide excision repair, p53 signaling pathway, pyrimidine metabolism, and spliceosome were enriched in the high *FOXD1* expression group (Figure 6).

## Discussion

Recently, *FOXD1* has been reported to be highly expressed in several cancers, such as colorectal cancer 17, lung cancer 18, breast cancer 19, and Hodgkin's lymphoma 22. Increased *FOXD1* expression has been shown to promote cell proliferation, migration, invasion, and tumorigenesis. However, the role of *FOXD1* in HNSCC has not been well characterized. The Cancer Genome Atlas (TCGA) was a large-scale effort to comprehensively characterize 33 major cancer types, including HNSCC, for which 528 cases were included 23. Our study using TCGA database showed that *FOXD1* mRNA expression was higher in HNSCC tissue than in normal tissue. The HNSCC mRNA microarray dataset GSE6631 was downloaded from the GEO database. The GEO database is a public functional genomics data repository that includes array- and sequence-based gene profiles and next-generation sequencing 24. Analysis of the GEO dataset showed that *FOXD1* was significantly upregulated in HNSCC tissues compared with corresponding adjacent normal tissues. Furthermore, we collected 162 paired HNSCC tissues and adjacent normal tissues to validate our bioinformatics analysis. Quantitative RT-PCR analysis confirmed that *FOXD1* mRNA was significantly overexpressed in HNSCC tissues. These findings indicated that *FOXD1* may function as an oncogene in HNSCC.

HNSCC is a heterogeneous group of tumors located in the upper aerodigestive tract with multifactorial etiologies. Alcohol consumption, betel nut chewing, and human papillomavirus infection are significant risk factors for development of HNSCC in the upper gastrointestinal tract 25-28. In contrast, tobacco smoking is a significant risk factor for development of HNSCC in the upper respiratory tract 29, 30. In the present study, we found that tumors located in the upper respiratory tract (larynx) showed significantly higher *FOXD1* expression levels than those in the upper gastrointestinal tract (oral cavity, oropharynx and hypopharynx), which suggested that laryngeal squamous cell carcinoma may be more susceptible to *FOXD1* overexpression.

Early diagnosis is critical to successful management of cancer 31, 32. More than half of HNSCC patients are at advanced stages at the time of diagnosis because of concealment of the anatomical site and lack of specific and reliable indicators. Furthermore, the age of diagnosis is slowly decreasing 33. Therefore, identification of biomarkers for early diagnosis of HNSCC is of great importance. In the current study, ROC curves were generated, and AUC values were calculated to evaluate *FOXD1* expression as a potential diagnostic marker of HNSCC. The AUC values of TCGA cohort and the validation cohort were 0.855 and 0.843, respectively. Compared to traditional tumor markers, such as CEA (Carcino Embryonic Antigen), SCC (Squamous Cell Carcinoma Antigen), TPS (Tissue Polypeptide Specific Antigen), and CYFRA 21-1 34, 35, *FOXD1* had a greater ability to discriminate patients with HNSCC from healthy individuals, which indicated that *FOXD1* could serve as a potential early diagnostic biomarker for HNSCC, especially when combined with other efficient markers.

Molecular characteristics, pathogenesis, and prognosis of head and neck cancers are heterogeneous. Several studies have shown that HPV-positive patients with HNSCC showed significantly improved overall and disease-free survival compared with HPV-negative patients 6, 36. Development of new technologies, such as microarray technology and next-generation sequencing, have allowed for collection of large amounts of data for molecular subclassification of cancer, which has allowed for development of individualized treatment programs 37, 38. Recent studies showed that overexpression of *FOXD1* were associated with poor OS in colorectal cancer 17, non-small cell lung cancer 18, and breast cancer 19. Consistent with these studies, the log-rank test performed in our study showed that high *FOXD1* expression was associated with significantly worse OS in both TCGA cohort and the validation cohort. In addition, multivariate proportional hazard Cox regression analysis showed that high *FOXD1* expression could serve as an independent indicator of poor prognosis in patients with HNSCC, which suggested that* FOXD1* may be a promising prognostic biomarker and therapeutic target for HNSCC. Recurrence rate is believed to be an important contributor to the poor prognosis associated with HNSCC 39. Approximately 30-40% of patients with HNSCC suffer from recurrence or metastasis following treatment 40. In our study, multivariate Cox regression analysis showed that *FOXD1* was an independent predictor of recurrence. This result suggested that monitoring *FOXD1* expression may improve outcomes in patients with HNSCC.

Cancer results from accumulation of genetic and epigenetic modifications of oncogenes and tumor-suppressor genes, resulting in metabolic dysfunction and uncontrolled proliferation 41, 42. Amplification of DNA is the major genetic change that results in cancer-specific expression of critical genes 43, 44. In addition, DNA methylation is an important epigenetic modification involved in the inactivation of numerous tumor suppressor genes 45, 46. Therefore, we explored the association of *FOXD1* expression with copy number alterations and promoter methylation levels to determine the mechanism of *FOXD1* overexpression in HNSCC. The results showed that DNA amplification was associated with elevated *FOXD1* RNA expression. In addition, we also found that* FOXD1* RNA expression was negatively correlated with promoter methylation level. These findings indicated that both genetic and epigenetic alterations contributed to dysregulation of* FOXD1* in HNSCC. Gene set enrichment analysis showed that bladder cancer, cell cycle, DNA replication, glycosaminoglycan biosynthesis chondroitin sulfate, homologous recombination, glycan biosynthesis, nucleotide excision repair, p53 signaling pathway, pyrimidine metabolism, and spliceosome may be key pathways regulated by *FOXD1* in HNSCC. These findings should be further validated by rigorous *in vitro* and *in vivo* experiments.

## Conclusion

In summary, our analysis showed that *FOXD1* expression was significantly elevated in HNSCC tissues relative to normal tissues in TCGA database, the GEO database, and the validation cohort. Genetic and epigenetic alterations contributed to upregulation of* FOXD1* in HNSCC. In addition, elevated* FOXD1* expression was a good diagnostic biomarker and independent predictor of poor OS and RFS in patients with HNSCC. Furthermore, *FOXD1* overexpression was significantly associated with bladder cancer, cell cycle, DNA replication, nucleotide excision repair, and p53 signaling pathways. Future studies are needed to characterize the specific role of *FOXD1* in HNSCC.

## Figures and Tables

**Table 1:** Association between *FOXD1* expression and clinicopathological features of patients with HNSCC in The Cancer Genome Atlas (TCGA) database. Bold font indicates statistically significant differences

Characteristics	N	Mean±SD	*P* value
**Gender**			
Female	136	7.372±1.556	0.814
Male	384	7.405±1.341	
**Age**			
<60y	233	7.379±1.360	0.761
≥60y	286	7.416±1.432	
Not available	1		
**Smoking history**			
No	117	7.745±1.336	0.003
Yes	391	7.306±1.400	
Not available	12		
**Alcohol history**			
No	162	7.356±1.504	0.66
Yes	347	7.415±1.365	
Not available	11		
**Histologic grade**			
G1+2	366	7.402±1.334	0.603
G3+4	132	7.330±1.578	
Not available	22		
**Tumor site**			
Oral cavity\Oropharynx\Hypopharynx	404	7.522±1.300	0.001
Larynx	116	9.961±1.631	
**HPV status**			
Negative	73	7.298±1.148	0.906
Positive	38	7.263±1.644	
Not available	409		
**Perineural invasion**			
Negative	193	7.376±1.410	0.087
Positive	169	7.614±1.204	
Not available	158		
**Pathologic tumor category**			
Tis/T1/T2	185	7.497±1.269	0.315
T3/T4	273	7.362±1.491	
Not available	62		
**Pathologic nodal category**			
No	176	7.261±1.345	0.062
Yes	244	7.518±1.455	
Not available	100		
**Pathologic stage**			
I+II	101	7.322±1.339	0.446
III+IV	347	7.444±1.438	
Not available	72		

**Table 2:** Association between *FOXD1* expression and clinicopathological features of patients with HNSCC patients in our validation cohort

Characteristics	N	Mean±SD	*P* value
**Gender**			
Female	37	0.091±0.073	0.794
Male	125	0.094±0.076	
**Age**			
<60y	73	0.100±0.083	0.300
≥60y	89	0.878±0.069	
**Smoking history**			
No	58	0.104±0.080	0.170
Yes	104	0.087±0.072	
**Alcohol history**			
No	105	0.088±0.074	0.237
Yes	57	0.103±0.076	
**Histologic grade**			
Well/moderately	112	0.098±0.077	0.296
Poorly	50	0.084±0.070	
**Tumor site**			
Oral cavity/Oropharynx/Hypopharynx	129	0.093±0.075	0.838
Larynx	33	0.096±0.078	
**Tumor invasion**			
Tis/T1/T2	61	0.096±0.068	0.732
T3/T4	101	0.092±0.080	
**Lymphatic metastasis**			
No	67	0.090±0.074	0.586
Yes	95	0.096±0.077	
**Clinical stage**			
I+II	77	0.088±0.067	0.361
III+IV	85	0.099±0.082	

**Table 3:** Univariate and multivariate Cox proportional hazard regression analysis of clinicopathologic features and *FOXD1* expression for overall survival. Bold font indicates statistically significant differences

Variables	Univariate analysis			Multivariate analysis		
	HR	95% CI	*P*	HR	95% CI	*P*
Age (≥ 60y vs. <60y)	1.318	1.003-1.731	0.045	1.042	0.736-1.476	0.815
Gender (Female vs. male)	1.349	1.014-1.796	0.040	0.957	0.647-1.417	0.827
Smoking history (Yes vs. No)	1.123	0.803-1.572	0.498			
Alcohol history (Yes vs. No)	0.942	0.709-1.252	0.680			
Histologic grade (G3/4 vs. G1/2)	0.867	0.637-1.180	0.363			
Perineural invasion (positive vs. negative)	2.135	1.516-3.007	1.42E-05	2.051	1.436-2.931	7.90E-05
Pathologic stage (III/IV vs. I/II)	1.754	1.203-2.558	0.004	1.739	1.051-2.877	0.031
*FOXD1* expression (High vs. Low)	1.665	1.264-2.194	2.93E-04	1.849	1.280-2.670	0.001

**Table 4:** Univariate and multivariate Cox proportional hazard regression analysis of clinicopathologic features and *FOXD1* expression for recurrence-free survival. Bold font indicates statistically significant differences

Variables	Univariate analysis			Multivariate analysis		
	HR	95% CI	*P*	HR	95% CI	*P*
Age (≥ 60y vs. <60y)	1.210	0.829-1.765	0.323			
Gender (Female vs. male)	1.006	0.654-1.546	0.979			
Smoking history (Yes vs. No)	0.959	0.622-1.480	0.851			
Alcohol history (Yes vs. No)	1.762	1.111-2.794	0.016	1.532	0.947-2.478	0.082
Histologic grade (G3/4 vs. G1/2)	0.753	0.481-1.180	0.216			
Perineural invasion (positive vs. negative)	1.517	0.968-2.378	0.069			
Pathologic Stage (III/IV vs. I/II)	2.398	1.303-4.411	0.005	2.285	1.237-4.220	0.008
*FOXD1* expression (High vs. Low)	1.725	1.152-2.583	0.008	1.650	1.058-2.575	0.027

**Table 5:** Gene set enriched in HNSCC samples with high* FOXD1* expression. NES: normalized enrichment score; NOM: nominal; FDR: false discovery rate

Gene set name	NES	NOM *p*-val	FDR *q*-val
KEGG_NUCLEOTIDE_EXCISION_REPAIR	2.068	< 0.001	0.020
KEGG_GLYCOSAMINOGLYCAN_BIOSYNTHESIS_CHONDROITIN_SULFATE	2.051	0.002	0.015
KEGG_CELL_CYCLE	2.047	0.002	0.011
KEGG_BLADDER_CANCER	1.952	< 0.001	0.032
KEGG_HOMOLOGOUS_RECOMBINATION	1.935	< 0.001	0.032
KEGG_N_GLYCAN_BIOSYNTHESIS	1.897	0.008	0.034
KEGG_DNA_REPLICATION	1.870	0.008	0.046
KEGG_PYRIMIDINE_METABOLISM	1.856	0.006	0.041
KEGG_SPLICEOSOME	1.837	0.018	0.045
